# On the pseudo-hyperbolic behavior of charge transfer resistance–temperature dependence in corrosion behavior of Nickel based glass alloy

**DOI:** 10.1038/s41598-022-10462-y

**Published:** 2022-04-19

**Authors:** Khadijah M. Emran, Inam M. A. Omar, Sanaa T. Arab, Noureddine Ouerfelli

**Affiliations:** 1 Chemistry Department, College of Science, Taibah University, Al-Madinah, Saudi Arabia; 2 Chemistry Department, Faculty of Sciences, Al-Faisaliah, King Abdulaziz University, Jeddah, Saudi Arabia; 3 Institut Supérieur des Technologies Médicales de Tunis, LR13SE07, Laboratoire de Biophysique et Technologies Médicales, Université de Tunis El Manar, Tunis, Tunisia

**Keywords:** Chemistry, Chemical engineering, Electrochemistry, Theoretical chemistry

## Abstract

Temperature plays an important role in promoting the corrosion of metals. The Arrhenius plot can interpret the corrosion rate-temperature dependence, where the Arrhenius behavior gives a geometrical meaning and makes explicit a positive or negative linear dependence of charge transitivity and temperature. In addition, according to the Arrhenius interpretation, it represents the energy that the molecule in the initial state of the process must acquire before it can take part in the reaction, whether it is a physical, or a chemical process. Taking into account the deviation from the linearity, we have extended the Arrhenius-type expression by one term in 1/*T*^2^ and we have given some physical meaning to the new related coefficients for which it is found that they depend closely on the number of acid hydrogen atoms in the polyacid for the corrosion and passivation of the Nickel based metallic glass alloy of the composition Ni_82.3_Cr_7_Fe_3_Si_4.5_B_3.2_. Moreover, we can consider that the deviation to the Arrhenius linear behavior as a super-Arrhenius behavior In addition, a mathematical analysis of the trend of experimental scatter points of the charge transfer resistance with temperature permits us to reveal an interesting homographic behavior which leads us to suggest an original empirical model with only two optimal adjustable parameters, as well as a new pseudo-power dependence of the number of hydrogen atoms in the polyacid.

## Introduction

Generally, in corrosive electrolytic solution, metals get oxidized at anode and charges (electrons) are transferred through electro-circuit. This process is known as charge transfer, and any resistance in the charge transfer process is known as charge transfer resistance (*R*_ct_). The Stern-Geary equation (describing the relationship between the polarization resistance (*R*_*p*_) and the corrosion current density (*i*_*corr*_).), which derived because of the mixed potential theory of Wagner and Traud, relates the corrosion current (corrosion rate) to the inverse of the resistance to charge transfer^1–5^. In other words, corrosion rate is also reflected in terms of corrosion current density (*i*_corr_). For this, corrosion rate can be quantitatively measured by means of polarization resistance techniques^2,6–9^.

In most chemical reactions, an increase in temperature is accompanied by an increase in reaction rate due to the increase of kinetic energy and speeds up the chemical reaction^10,11^, and the corrosion reaction rate doubles for each 10 °C rise in temperature. Therefore, it is important to take into consideration the influence of temperature when analyzing why metals corrode. The effect of temperature on the corrosion rate can be analyzed in terms of activation energy from Arrhenius behavior for the overall corrosion process^12–18^.

If the corrosion rate is only controlled by the metal oxidation process, then the corrosion rate will increase exponentially with an increase in temperature following an Arrhenius relationship. The equation was first proposed by Van’t Hoff (1884), in 1889 Svante Arrhenius provided a physical justification and interpretation for it^19,20^.

Generally, for the majority of experimenters, the charge transfer resistance–temperature dependence is treated by linear regression of the Arrhenius behavior by the plot of the logarithm of the charge transfer resistance versus the reciprocal of absolute temperature.

Nevertheless, we have noted that there is some feeble deviation to the linearity for numerous works, which exceed the experimental errors bar. To fix this observation we will proceed in two manners in the present work.

(i) we will extend the Arrhenius type-equation by a supplementary term in (1/*T*^2^) to reduce the discrepancy with the experience and test the deviation to the Arrhenius linearity if it can be classified as a sub-Arrhenius or super-Arrhenius behavior for the studied system. In addition, we will model the effect of the protons number in polyacid on the Arrhenius parameters.
(ii) Regarding the trend of experimental scatter points of the charge transfer resistance with temperature that likes a hyperbolic behavior, we will mathematically investigate this variation and suggest an original homographic model with only two optimal adjustable parameters for which we try to give some physical significance.

We note the present work is a theoretical continuation and modeling of our principally experimental previous work results^21^ where all experimental data are presented and discussed in terms of apparent activation energies, active, and passive, as well as the enthalpies and entropies of the dissolution process.

## Materials and methods

The material chosen for the study was Nickel based metallic glass alloy of the composition Ni_82.3_Cr_7_Fe_3_Si_4.5_B_3.2_ weight percent (*wt*%) from Vacuumschmelze. The experimental data of our previous paper^21^ on the effect of temperature ranging from (20 to 80) °C on corrosion and the electrochemical behavior of the glassy alloy in HCl, H_2_SO_4_, and H_3_PO_4_ were used in this study. Full details are described in previous paper^21^.

## Deviation to the Arrhenius-type equation

### Arrhenius-type equation

Activation energy (*E*_*a*_) is strictly combined with kinetics of chemical reactions. The effect of temperature on reaction rates is calculated using the Arrhenius equation. A common sense is that at higher temperatures, the reactants molecules colliding probability becomes higher and the reaction proceeds faster. The bond cleavages and rearrangements of molecules generally increase as temperature rises. This is through the collision theory, transition state theory or a chemical reaction.

The Swedish chemist Svante Arrhenius was the first who combined the concepts of activation energy and the Boltzmann distribution law into one relationship. This described as the Arrhenius equation. The familiar Arrhenius equation, Eq. (1) is given as follows^20^:

1 $${R}_{ct}={A}_{ct}{e}^{\frac{{E}_{a}}{RT}}$$

where *Ea* is the activation energy, *A*_ct_ is the pre-exponential factor which, is also known as the frequency factor, and it mathematically represents the limiting theoretical value of the charge transfer resistance at infinite temperature. This factor is also interpreted as an entropic factor which, it is presenting the frequency of collisions between reactant molecules at a standard concentration. Noteworthy, although *A*_ct_ is often described as temperature independent, but it is actually dependent on it. This is because *A*_ct_ is related to molecular collision, which is a function of temperature.

These two Arrhenius parameters are generally supposed both constants practically independent of temperature. The logarithm form of the preceding equation can be expressed as follows:

2 $$\mathrm{ln}{R}_{ct}=\mathrm{ln}{A}_{ct}+\frac{{E}_{a}}{R}\times \frac{1}{T}$$

**Figure 1:**
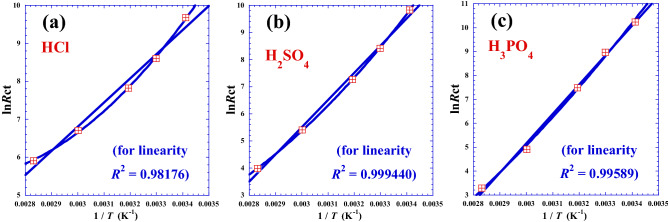
Variation of the logarithm of charge transfer resistance (ln*R*_ct_) as a function of the inverse of the reciprocal temperature (1/*T*) related to the hydrochloric acid (HCl), the sulfuric acid (H_2_SO_4_) and the phosphoric acid (H_3_PO_4_) for the systems at the temperature range: 20–80 °C; (Table 1). Straight lines: linear regression (Eq. 2). Curved lines: nonlinear regression (Eq. 4) with second degree of polynomial.

The plot of (ln*R*_ct_) as a function of the reciprocal of absolute temperature (1/*T*) gives approximately a handy straight line (Fig. 1a, b and c) whose slope is equal to (*Ea*/*R*) and the intercept on the ordinate is equal to (ln*A*_ct_). The results of the linear regression for the three acids are presented in the Table 1.

**Table 1:** Optimal Arrhenius parameters (*Ea* and ln*A*_ct_), Arrhenius temperature (*T*_*A*_ = –*Ea*/(*R.*ln*A*_ct_)), pre-exponential factor (*A*_ct_), and the entropic factor of Arrhenius – *R*·ln(*A*_ct_) from linear regression of Eq. (2).

Acid	ln*A*_ct_	*E*_a_	*T*_A_	*A*_ct_	– *R*·ln*A*_ct_	Δ*H*	Δ*S*	*R*-square
	–	kJ·mol^-1^	K	Ω·cm^2^	J·K^-1^·mol^-1^	kJ·mol^-1^	J·K^-1^·mol^-1^	–
HCl	− 12.310	52.976	517.59	4.5065 × 10^–6^	102.35	50.280	− 151	0.98176
H_2_SO_4_	− 24.546	83.287	408.10	2.1868 × 10^–11^	204.09	79.180	− 52	0.99440
H_3_PO_4_	− 31.733	102.27	387.61	1.6540 × 10^–14^	263.84	103.04	19	0.99589

Δ*H* and Δ*S* represents, respectively, the activation enthalpy and entropy determined from polarization and impedance measurements in previous work^21^.

**Figure 2:**
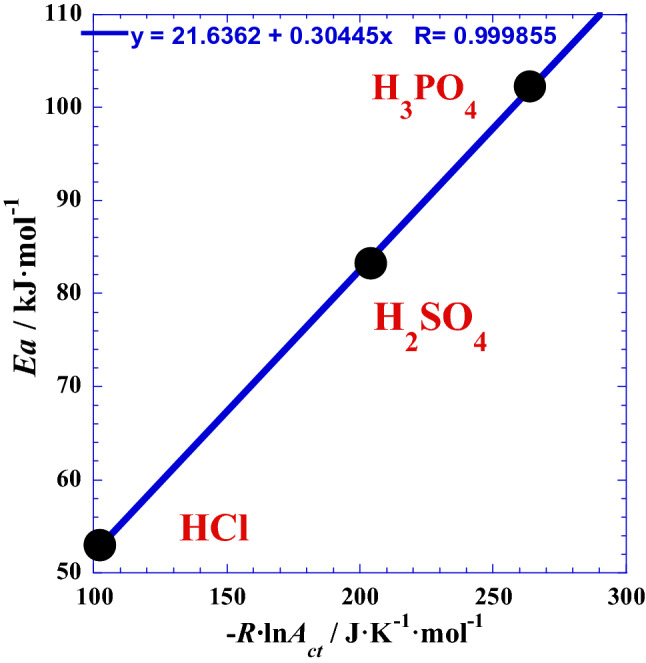
Correlation between the activation energy *Ea* (kJ/mol) from polarization and impedance measurements^21^ and the entropic factor of Arrhenius – *R*·ln(*A*_ct_/ Ω·cm^2^) / (J/K·mol) for the three acids HCl, H_2_SO_4_ and H_3_PO_4_ at atmospheric pressure.

Observing the variation of the two Arrhenius parameters (*Ea* and ln*A*_ct_) which are in the opposite senses (Table 1), we have thought about inspecting their mutual dependence^22^ by plotting one parameter against the second for the three studied polyacids H_*x*_B. In fact, the Fig. 2 shows interesting causal correlation for which the quasi-linearity inter-dependence can be expressed as follows:

3 $$Ea={E}_{a0}+{\tau }_{0}\cdot (- R\mathrm{ln}{A}_{ct})$$

where *E*_a0_ (= 21.64 kJ·mol^-1^) is the activation energy corresponding the null value of the entropic factor (i.e. at the limiting case of absence of any acidic proton), and the slope *τ*_0_ (= 304.45 K) is equivalent to an absolute temperature characteristic of the studied system at such conditions, and which can be also named as current Arrhenius temperature^22^. Moreover, we notice that (*τ*_0_) has an ambient value (31.3 °C). We can give then an inspiration for future investigators to think about the probable optimal value of working temperature.

**Figure 3:**
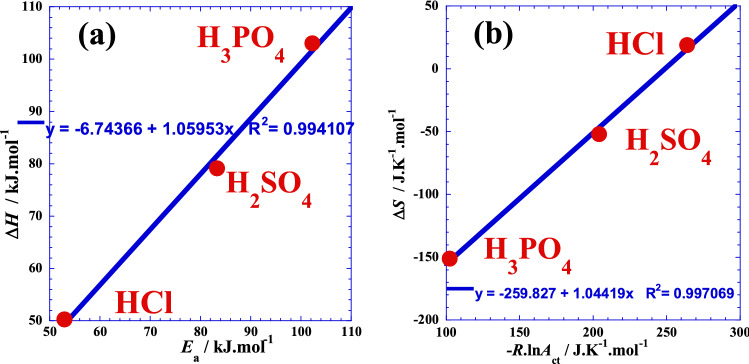
Correlation between the entropic factor of Arrhenius – *R*·ln(*A*_ct_/Ω·cm^2^) / (J/K·mol) and the thermodynamic parameters for glassy Ni_82.3_Cr_7_Fe_3_Si_4.5_B_3.2_ alloy corrosion; (**a**): with the activation enthalpy Δ*H* (kJ/mol) and (**b**): with the activation entropy Δ*S* (J/K·mol) determined from impedance measurements in previous work^21^ for the three polyacids HCl, H_2_SO_4_ and H_3_PO_4_ at atmospheric pressure.

We can conclude that there is a close causal correlation (Fig. 3) between the Arrhenius parameters (*Ea* and – *R*·ln(*A*_ct_)) and the thermodynamic parameters (Δ*H* and Δ*S*), respectively, for glassy Ni_82.3_Cr_7_Fe_3_Si_4.5_B_3.2_ alloy corrosion. In the same context, to confirm that the entropic factor of Arrhenius – *R*·ln(*A*_ct_) is equivalent to an entropy^22^, the Fig. 3b shows a clear causal correlation between the activation entropy Δ*S* (J/K·mol) determined from impedance measurements in our previous work^21^ and the logarithm of the entropic factor of Arrhenius – *R*·ln(*A*_ct_/ Ω·cm^2^) / (J/K·mol) for the three polyacids HCl, H_2_SO_4_ and H_3_PO_4_ at atmospheric pressure. Moreover, the slopes values of the two straight lines of Fig. 3 are very near the unit (1.06 and 1.04) which means that (*Ea* and Δ*H*) and (– *R*·ln(*A*_ct_) and Δ*S*) have approximately the same value of gap or “jump” when the number of protons (*x*) of the acid changes by one unit. We add that the Arrhenius parameters represent the movement between two energy levels related to transition state, while the thermodynamic parameters, as a state functions, represent the movement between two energy levels related to equilibrium thermodynamic states.

The highest *R*-square value (Table 1, Fig. 1c) indicates that in the case of triacid (H_3_PO_4_) the temperature dependence practically follows the Arrhenius behavior. Nevertheless, in the case of monoacid (HCl), we observe clear discrepancy with the Arrhenius linearity. In addition, the deviation of experimental points (Fig. 1a) to the straight line is systematic in nature and not random, which shows that the phenomenon, represented by variation of the logarithm of charge transfer resistance (ln*R*_ct_) as a function of the inverse of the reciprocal temperature (1/*T*), is not linear and we should extend the Arrhenius-type equation or think about another non linear model. In the following sections, we will present a new empirical expression reducing to a large degree the discrepancy with the experimental data points.

### Extended Arrhenius-type equation

Observing the trend of points in Fig. 1, we clearly see that there is feeble net deviation from the linearity of the Arrhenius behavior. For that, we suggest, as optimization by nonlinear regression, to fit simply the experimental results (ln*R*_ct_) *vs*. (1/*T*) with only a polynomial of second degree, which it can be expressed as follows:

4 $$\mathrm{ln}{R}_{ct}={a}_{0}+{a}_{1}\times \left(\frac{1}{T}\right)+{a}_{2}\times {\left(\frac{1}{T}\right)}^{2}$$

where *a*_i_ are optimal adjustable parameters. To give certain physical meaning of these parameters, the Eq. (4) can be re-expressed as follows:

5 $$\mathrm{ln}{R}_{ct}=\mathrm{ln}{A}_{0}-\frac{{E}_{1}}{R}\times \left(\frac{1}{T}\right)+\frac{{{E}_{2}}^{2}}{{R}^{2}}\times {\left(\frac{1}{T}\right)}^{2}$$

We note that due to the concavity of the curves in Fig. 1, the second term of Eq. (4) must be negative, that is why we have inserted a minus sign in Eq. (5) to have positive physical parameters. Table 2 presents values of the corresponding parameters.

**Table 2:** Optimal Arrhenius parameters (*E*_*i*_ and ln*A*_0_) from non-linear regression of Eq. (2).

Acid	ln*A*_0_	*E*_1_	*E*_2_	*R*-square
	–	kJ·mol^-1^	kJ·mol^-1^	–
HCl	38.2266	217.76	18.999	0.99929
H_2_SO_4_	19.708	153.79	17.779	0.99987
H_3_PO_4_	− 2.1065	56.450	14.547	0.99753

To help giving preliminary physical significances to the parameters of Eq. (5), we have tested the Eq. (3) on the parameters *E*_1_ and ln*A*_0_, we find practically the same behavior depicted in Fig. 2 where the slope (*τ*_0_ = 482.66 K) a current Arrhenius temperature with a *R*-square 0.9949.

We can notice an improvement of the correlation coefficient (*R*-square) proving that there effectively is deviation from the linearity of the Arrhenius behavior. Nevertheless, we observe the reverse case about the classification of the goodness of fit comparing with the results of Table 1 (i.e. in the Table 2, the quality is the best in the case of the mono acid HCl) we can attribute this effect to the conflict between the amount and parity of the chosen polynomial degree and, the experimental bar error of each acid, where the triacid (H_3_PO_4_) has two corresponding weak acids (H_2_PO_4_^-^ and HPO_4_^2-^). We note that the width of studied temperature range play also an effective role for this fact.

However, some researchers^23–28^ interpret the sign of the deviation to the Arrhenius linearity as a sub-Arrhenius or super-Arrhenius behaviors. For this fact, we remember that the activation energy *Ea* (Table 1) can be interpreted as a potential energy barrier which is assumed to be dependent on temperature *Ea*(*T*) and can be in general expressed at constant pressure as follows:

6 $${E}_{a}(T) = {\left(\frac{\partial (\mathrm{ln}{R}_{ct})}{\partial (1/RT)}\right)}_{P}$$

We note that in our case of polynomial form (Eq. 5) the activation energy *Ea*(*T*) can be expressed as follows:

7 $${E}_{a}\left(T\right)= {{2E}_{2}}^{2}\times \left(\frac{1}{RT}\right)- {E}_{1}$$

The positive slope (${{2E}_{2}}^{2}$) in Eq. (7), confirmed by the positive concavity of curvature indicated in Fig. 1, leads us to conclude that we are in the case of super-Arrhenius behavior.

On the other hand, the deviation to the classic Arrhenius linearity can be more interpreted by the *d*-exponential function’s flexibility^23–28^ in the deformed Arrhenius equation (Eq. 8) for the charge resistance–temperature dependence.

8 $${E}_{a}\left(T\right)= {{A}_{ct}\left[1+d \cdot \frac{{E}_{0}}{RT}\right]}^{d}$$

where (*E*_0_) is the height or the amount of the potential energy barrier and (*d*) is known as the deformation parameter for which the sign will indicate the nature of deviation to the Arrhenius linearity. The advantage of this deformed Arrhenius equation is that we can discuss the rate constant in terms of a single parameter^23–28^. The expression of Eq. (8) represents both the Arrhenius and non-Arrhenius behaviors of charge transfer resistance *R*_*ct*_(*T*). For positive values of (*d*), super-Arrhenius behavior is observed while for negative values of (*d*), sub-Arrhenius is observed. For values of d neighboring 0, we observe the classical Arrhenius behavior (Eq. 1) and the *E*_0_-value tends to the Arrhenius activation energy *Ea* (Table 1) Then, we can mathematically demonstrate the following expression:

9 $${E}_{a}\left(T\right)= \underset{d\to 0}{\mathrm{lim}}{{A}_{ct}\left[1+d \cdot \frac{{E}_{0}}{RT}\right]}^{d}= {A}_{ct}{e}^{\frac{{E}_{0}}{RT}}$$

For which we re-find the Eq. (1) form.

Moreover, for very feeble deviation, where the d-values are small, we can write the Eq. (6) (in accordance with (Eq. 8) in asymptotic expansion and, as a limit of succession, we can approximately write the following expression:

10 $${E}_{a}\left(T\right)\approx {E}_{0}+ d\cdot \frac{{{E}_{0}}^{2}}{RT}$$

The Eq. (10) can be reformulated in terms of the reciprocal of *Ea*(*T*) (i.e. γ(*T*) = 1/*Ea*(*T*)) as follows:

11 $$\gamma \left(T\right)\approx \alpha - d\cdot \frac{1}{RT}$$

where *α* is the inverse of Arrhenius-Eyring energy obstacle to charge transfer^23–28^. Table 3 presents the optimal values of linear regression of Eq. (11).

**Table 3:** Optimal adjustable parameters (*α* and *d*) from linear regression of Eq. (11).

Acid	*α*	*d*	*R*
	mol·kJ^-1^	–	–
HCl	0.1416	0.31964	0.99929
H_2_SO_4_	0.04882	0.09689	0.99987
H_3_PO_4_	0.025334	0.041189	0.99753

Positive values of *d* shown in Table 3 confirm that we are in the super-Arrhenius behavior where the activation energy *Ea*(*T*) increases with (1/*T*). We can also see that the very small value of (*d*) in the case of (H_3_PO_4_) confirms the feeble discrepancy with classical Arrhenius behavior indicated in Fig. 1c. We note that the value of (*d*) bears the same sign as the derivative of the activation energy *Ea*(*T*) with respect of the reciprocal of absolute temperature at constant pressure: ${\left(\frac{\partial {E}_{a}(T)}{\partial (1/T)}\right)}_{P}$ or the second derivative of the charge transfer resistance (*R*_ct_) with respect of the reciprocal of absolute temperature at constant pressure: ${\left(\frac{{\partial }^{2}{R}_{ct}(T)}{\partial {(1/T)}^{2}}\right)}_{P}$.

### Effect of the acid proton number on the model parameters

**Figure 4:**
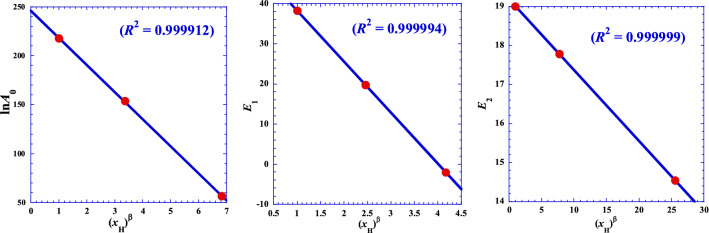
Variation of the three adjustable parameters (*E*_*i*_ and ln*A*_0_) of Eq. (5) as a function of the number of hydrogen (*x*_H_) of the acid.

The Table 2 shows that all of the three parameters of the Eq. (5) both decrease in a strictly monotonous way, which it inspires us to investigate possible relationship with the number of hydrogen (*x*_H_) of the acid. Some different simulations for reducing the line’s curvature lead us to obtain the best linearization form expressed as follows:

12 $$Y={Y}_{0}(1-\delta \cdot {x}_{H}^{\beta })$$

where *Y* is one of the three parameters (*E*_1_, *E*_2_ and ln*A*_0_) of Eq. (5) and, *Y*_0_, *δ* and *α* are three adjustable parameters. Results of this finding are shown by Fig. 4 and presented in Table 4.

**Table 4:** Optimal Arrhenius parameters (*Y*_0_, *δ* and *β*) from non-linear regression of Eq. (12).

Parameter	*Y*		
	ln*A*_0_	*E*_1_	*E*_2_
*β*	1.30	1.75	2.95
*δ*	0.1125	0.2495	0.00945
*Y*_0_	245.94	50.975	19.180
*R*-square	0.999912	0.999994	0.999999

This curious and interesting pseudo-power behavior of Eq. (12) leads us to give up the Arrhenius-type model and think about another mathematical form like the hyperbolic behavior.

**Figure 5:**
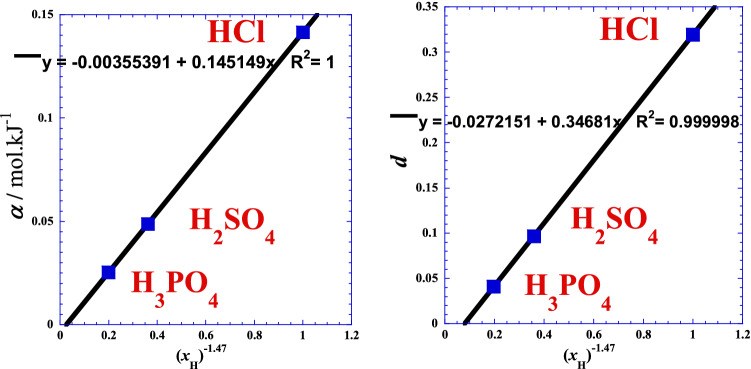
Variation of the two optimal adjustable parameters (*α* and *d*) from linear regression of Eq. (11) as a function of the number of hydrogen (*x*_H_) of the acid.

In the same context, we have also done some different simulations for reducing the line’s curvature which lead us to obtain the best linearization form for the two optimal adjustable parameters (*α* and *d*) of Eq. (11) (Fig. 5). We found that similar pseudo-power behavior obeying Eq. (12) is observed for *β* = – 0.147, both for the two parameters (*α* and *d*) with excellent correlation coefficients (Fig. 5).

We conclude that the number of hydrogen (*x*_H_) of the polyacid has an important effect, which can be quantified and modeled for eventual future estimation, or prediction and can open window to improve or develop some theories.

## Pseudo-hyperbolic behavior of charge transfer resistance with temperature

**Figure 6:**
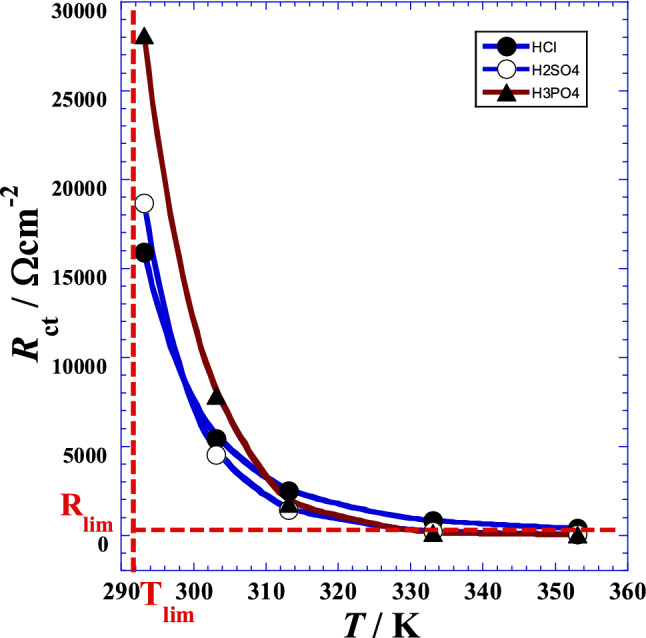
Variation of the charge transfer resistance (*R*_ct_) as a function of temperature (*T*) for different acids. (●): HCl; (○): H_2_SO_4_; (▲): H_3_PO_4_.

The graphical representation (Fig. 6) of the charge transfer resistance (*R*_ct_) as a function of temperature (*T*) shows a pseudo-hyperbolic behavior characterized by the tendency towards very low values when the temperature increases and a tendency towards very high values when the temperature decreases. To analyze this behavior, we will perform two linearization tests.

Nevertheless, in the case where the pseudo-hyperbolic behavior is followed, the value of the limiting temperature (*T*_lim_) related to the vertical asymptote when (*R*_ct_) tends to infinity and the value of the limiting charge transfer resistance (*R*_lim_) when the temperature (*T*) tends to infinity, are unknown. For that, we will proceed to two linearization forms. We add that before finding these two linearization forms, we have tested graphically several different transformed independent-dependent variables dependencies, for which we can cite for example *R*_ct_ = *f*(1/*T*), 1/*R*_ct_ = *f*(*T*), 1/*R*_ct_ = *f*(1/*T*) and *R*_ct_·*T* = *f*(*T*). All these mathematical functions exhibit non-linear behavior. Nevertheless, we find only two further expressions {*T*·*R*_ct_ = *f*(*R*_ct_) and *T*·(*R*_ct_ + *R*_1_) = *f*(*R*_ct_)} showing a good linearity which we will develop in the following section.

### First linearization form

**Figure 7:**
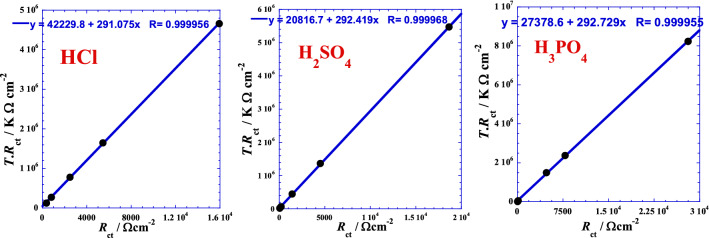
Linear correlation between the product temperature-charge transfer resistance *T*·*R*_ct_ and the charge transfer resistance *R*_ct_ for each acid.

This form consists to test the linearization in the positive branch of the prospective parabola by testing the product *T*·*R*_ct_ as a function of *R*_ct_ to situate approximately the position and arrangement of the curve. The following figures (Fig. 7) show an excellent apparent linearity that can be expressed as follows:

13 $${TR}_{ct}= {a}_{0}+ {a}_{1}\cdot {R}_{ct}$$

Taking into account the dimensional equations, we set *a*_1_ = *T*_0_ and *a*_0_ = *T*_0_.*R*_0_. We then obtain a more significant hyperbolic form, which can be expressed as follows:

14 $${R}_{ct}= \frac{{R}_{0}{T}_{0}}{T {- T}_{0}}$$

where *R*_0_ and *T*_0_ are two optimal adjustable parameters, which are equivalent to a charge transfer resistance and temperature, respectively. Values of these parameters are given in the Table 5. However, the *R*_0_–value mathematically represents the value that the charge transfer resistance can take, when the temperature (*T*) reaches twice the value of *T*_0_ while the Fig. 6 and Table 5 indicates a certain discrepancy leading us to speculate that the real phenomenon is probably described by a more complexing parabola or another mathematical form which is consolidated by the fact that the limiting temperature value (*T*_0_) is very near to the minimum temperature (293.15 K) of the investigated temperature range. On the other hand, at very high temperature, the model of Eq. (14) indicates that the charge transfer resistance tends to a very low value, showing that the corrosion is accentuated.

**Table 5:** Optimal adjustable parameters’ values of Eq. (14), for each acid.

Acid	*T*_0_	*T*_0_	*R*_0_	*R*-square
	K	°C	Ω cm^2^	–
HCl	291.08	17.93	145.08	0.999912
H_2_SO_4_	292.42	19.27	71.19	0.999936
H_3_PO_4_	292.73	19.58	93.53	0.999912

### Second linearization form

**Figure 8:**
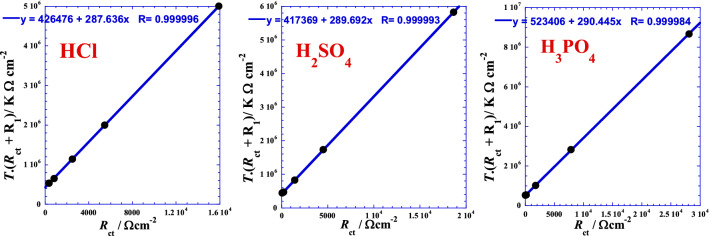
Linear dependence between the product *T*.(*R*_ct_ + *R*_1_) of Eq. (15) and the charge transfer resistance *R*_ct_ for each acid.

This form consists to test the linearization in the negative branch of the prospective parabola by testing the product *T*·(*R*_ct_ + *R*_1_) as a function of *R*_ct_ to situate approximately the position and arrangement of the curve and its asymptotes and where *R*_1_ is a free adjustable parameter. The following figures (Fig. 8) show an excellent apparent linearity, which can be expressed as follows:

15 $${T(R}_{ct}+{R}_{1})= {a}_{0}+ {a}_{1}\cdot {R}_{ct}$$

Taking into account the dimensional equations, we set *a*_1_ = *T*_0_ and *a*_0_ = *T*_0_.*R*_0_. We then obtain a more significant homographic form, which can be expressed as follows:

16 $${R}_{ct}= \frac{{R}_{0}{T}_{0}{-R}_{1}\cdot T}{T {- T}_{0}}$$

where *R*_0_, *R*_1_ and *T*_0_ are three optimal adjustable parameters, which are equivalent to two charge transfer resistances and temperature, respectively. Values of these parameters are given in the Table 6.

**Table 6:** Optimal adjustable parameters’ values of Eq. (16) for each acid.

Acid	*T*_0_	*T*_0_	*R*_0_	*R*_1_	*R*-square
	K	°C	Ω cm^2^	Ω cm^2^	–
HCl	287.64	14.49	1482.69	1150	0.999992
H_2_SO_4_	289.69	16.54	1440.73	1125	0.999986
H_3_PO_4_	290.45	17.30	1802.08	1500	0.999968

The temperature limit values are lower than that of the first linearization, which shows that this technique has given better results. It can then be considered that this limiting temperature (*T*_0_) corresponds to a very high value of the charge transfer resistances (Fig. 6) where the corrosion is practically slowed down.

**Figure 9:**
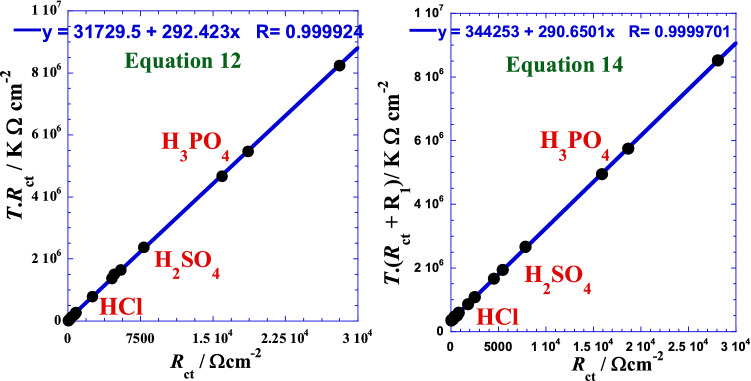
Linear correlation between the product temperature-charge transfer resistance *T*·*R*_ct_ of Eq. (13) or *T*.(*R*_ct_ + *R*_1_) of Eq. (15) and the charge transfer resistance *R*_ct_ for overall the three acids.

Finally, we note that though the two homographic proposed models give specific parameters values of each acid, we have found that we can treat overall the three acids in the same time by only global parameters with reliable accuracy. Then, we obtain excellent linearization shown by (Fig. 9) for which, each the corresponding global optimal adjustable parameters are given by Table 7. It’s interesting to inspect in future the variation of these parameters for other systems in different conditions to more reveal their physical meaning.

**Table 7:** Optimal adjustable parameters’ values of the two hyperbolic suggested models Eqs. (14), and (16) for overall the three acids.

Model	*T*_0_	*T*_0_	*R*_0_	*R*_1_	*R*-square
	K	°C	Ω cm^2^	Ω cm^2^	–
Equation 14	292.42	19.27	108.51	0	0.999849
Equation 16	290.65	17.50	1184.42	960	0.999940

### Arrhenius activation energy

We note that we can estimate an average value of the Arrhenius activation energy from the Eq. (14) by considering that the linear Arrhenius behavior is not followed; and the Arrhenius-type equation (Eq. 2) must be expressed as follows:

17 $$\mathrm{ln}{R}_{ct}=\mathrm{ln}{A}_{ct}(T)+\frac{{E}_{a}(T)}{R}\left(\frac{1}{T}\right)$$

where the Arrhenius parameters *A*_ct_ and *E*_*a*_ become dependent of temperature. For that to determine these parameters, we must consider the general definitions of Eq. (6) and not the slope of linear regression.

18 $${E}_{a}(T) = R{\left(\frac{\partial (\mathrm{ln}{R}_{ct})}{\partial (1/T)}\right)}_{P}$$

So, using Eqs. 14 and 18, we can obtain the following expression of the dependence of activation energy with temperature.

19 $$\frac{{E}_{a}(T)}{R}=\frac{{T}^{2}}{T{-T}_{0}}$$

Nevertheless, this expression can be transformed as follows to be easily integrated later for statistical handling:

20 $$\frac{{E}_{a}(T)}{R}=T{+ T}_{0} +\frac{{{T}_{0}}^{2}}{T{-T}_{0}}$$

To give an approximate estimation of the mean activation energy *Ea* that should be obtained by classical linear regression we can apply the following reasoning. We can calculate the average value of the function *Ea*(*T*) expressed by Eq. (20) over a temperature interval [*T*_min_, *T*_max_] such as in our situation; [293.15,353.15]K, by the following expression:

21 $$\overline{{E}_{a}}=\frac{1}{{T}_{max}-{T}_{min}}{\int }_{{T}_{min}}^{{T}_{max}}{E}_{a}(T)dT$$

Which it can be adapted for Eq. (20) and can lead to a practical expression (Eq. 22) for an average value of activation energy without using direct linear regression of ln*R*_ct_ with 1/*T*.

22 $$\overline{{E}_{a}}=R\left[{T}_{0}+\frac{{T}_{max}+{T}_{min}}{2}+\frac{{{T}_{0}}^{2}}{{T}_{max}-{T}_{min}}ln\left(\frac{{T}_{max}-{T}_{0}}{{T}_{min}-{T}_{0}}\right)\right]$$

Nevertheless, we must notice that the small number of experimental data points and the existence of the corresponding errors can result in an average value of activation energy with a relative deviation about 10% or more.

## Conclusion

Generally, for the majority of experimenters, the charge transfer resistance–temperature dependence is treated by linear regression of the Arrhenius behavior by the plot of the logarithm of the charge transfer resistance versus the reciprocal of absolute temperature.

Nevertheless, we have noticed that there is some feeble deviation to the linearity for numerous works, which exceed the experimental errors bar. To fix this observation and reduce the discrepancy between experimental and calculated values, we have proceeded in two manners.

(i). We have extended the Arrhenius type-equation by a supplementary term in (1/*T*^2^) to reduce the discrepancy with the experience and classify the nature of deviation as a super-Arrhenius behavior showing that the effective activation energy increases when the temperature decreases.
(ii). Regarding the trend of experimental scatter points of the charge transfer resistance with temperature, which likes a hyperbolic behavior, we have mathematically investigated this variation and suggested an original homographic model with only two optimal adjustable parameters for which we gave some physical meaning. In the same context, we have suggested a mathematical formula allowing to indirectly calculate the familiar Arrhenius activation energy using only the parameters of the homographic model for each acid or for overall the three acids.
(iii). In addition, in the present work, we discover interesting property, a causal correlation with the number of protons of polyacid, which can open a new theoretical field and encourage experimenters to validate this finding by the use of other acids in future investigation on different systems.
